# Stroke and Risks of Development and Progression of Kidney Diseases and End-Stage Renal Disease: A Nationwide Population-Based Cohort Study

**DOI:** 10.1371/journal.pone.0158533

**Published:** 2016-06-29

**Authors:** Chia-Lin Wu, Chun-Chieh Tsai, Chew-Teng Kor, Der-Cherng Tarng, Ie-Bin Lian, Tao-Hsiang Yang, Ping-Fang Chiu, Chia-Chu Chang

**Affiliations:** 1 Division of Nephrology, Department of Internal Medicine, Changhua Christian Hospital, Changhua, Taiwan; 2 School of Medicine, Chung-Shan Medical University, Taichung, Taiwan; 3 Institute of Clinical Medicine, National Yang-Ming University, Taipei, Taiwan; 4 Internal Medicine Research Center, Changhua Christian Hospital, Changhua, Taiwan; 5 Division of Nephrology, Department of Medicine, Taipei Veterans General Hospital, Taipei, Taiwan; 6 Department and Institute of Physiology, National Yang-Ming University, Taipei, Taiwan; 7 Graduate Institute of Statistics and Information Science, National Changhua University of Education, Changhua, Taiwan; 8 Environmental and Precision Medicine Laboratory, Changhua Christian Hospital, Changhua, Taiwan; University of Sao Paulo Medical School, BRAZIL

## Abstract

**Background:**

There is little information about the association between stroke and kidney diseases. We aimed to investigate the impact of stroke on long-term renal outcomes.

**Methods:**

In this large population-based retrospective cohort study, we identified 100,353 subjects registered in the National Health Insurance Research Database of Taiwan from January 1, 2000, through December 31, 2012, including 33,451 stroke patients and 66,902 age-, sex- and Charlson’s comorbidity index score-matched controls.

**Results:**

The incidence rate of chronic kidney disease (CKD) was higher in the stroke than in the control cohort (17.5 *vs*. 9.06 per 1000 person-years). After multivariate adjustment, the risk of developing CKD was significantly higher in patients with stroke (adjusted hazard ratio [aHR] 1.43, 95% confidence interval [CI] 1.36–1.50, *P*<0.001). Subgroup analysis showed that stroke patients <50 years (aHR 1.61, *P*<0.001) and those with concomitant diabetes mellitus (aHR 2.12, *P*<0.001), hyperlipidemia (aHR 1.53, *P*<0.001) or gout (aHR 1.84, *P*<0.001) were at higher risk of incident CKD. Additionally, the risks of progression to advanced CKD and end-stage renal disease (ESRD) were significantly higher for stroke patients (aHRs, 1.22 and 1.30; *P* = 0.04 and *P* = 0.008, respectively), independent of age, sex, comorbidities and long-term medications.

**Conclusions:**

Stroke is associated with higher risks for incident CKD, decline in renal function and ESRD. Younger stroke patients, as well as those with concomitant diabetes mellitus, hyperlipidemia or gout are at greater risk for kidney diseases.

## Introduction

Stroke is becoming an important health issue as populations are ageing rapidly worldwide. In Taiwan, for example, 11.5% of the population in 2012 was >65 years old [1], and stroke is the third most frequent cause of death, placing a substantial burden on the national healthcare system [2]. Although the age-standardized rates of stroke mortality decreased worldwide from 1990 to 2010, the absolute numbers of people having strokes every year increased; moreover, the numbers of stroke survivors, stroke-related deaths, and years lived with disability are large and increasing [3]. The incidence of age-standardized annual first-ever stroke is higher in Chinese than Caucasians [4].

Chronic kidney disease (CKD) is another important cause of years lived with disability, with its incidence increasing approximately 50% from 1990 to 2013 [5]. Taiwan has the highest prevalence and the second-highest incidence rates of end-stage renal disease (ESRD) throughout the world, with rates largely attributed to the progression of CKD [6].

Stroke and CKD share similar cardiometabolic risk factors [7]. The brain and kidneys have similar anatomical and functional vasoregulation of microvasculature [8]. Both organs have low vascular resistance systems allowing continuous high-volume perfusion [9]. The association between the brain and kidneys remains unclear, though previous studies show that CKD was associated with cardiovascular and cerebrovascular diseases [7, 10]. Cerebrovascular diseases may also affect renal diseases, inasmuch as stroke may be associated with CKD or ESRD [11–13]. However, these studies were either uncontrolled or small in sample size with short-term follow-up. The cause and effect relationships between stroke and CKD remain unclear, especially whether these relationships are bidirectional.

To our knowledge, the impact of stroke on the risk of the full spectrum of CKD has not been thoroughly examined. This retrospective study, involving a large-scale nationwide cohort, evaluated the effects of stroke on the development and progression of CKD and ESRD.

## Materials and Methods

### Data Source

Data were retrieved from the Taiwan National Health Insurance Research Database (NHIRD), which includes all claims data from the National Health Insurance program. These claims include demographic data, ambulatory care, records of clinic visits, hospital admissions, dental services, prescriptions and disease status. The National Health Insurance program, which was started in Taiwan in March 1995, covers >99% of the total population, or approximately 23 million people. Diagnostic codes for identifying diseases were based on International Classification of Diseases, Ninth Revision, Clinical Modification (ICD-9-CM). Use of ICD-9-CM codes in the NHIRD has shown high accuracy and validity [14–16]. Because de-identified and encrypted secondary data were analyzed, this study was exempted from full review and approved by the Institutional Review Board of the Changhua Christian Hospital (approval number 150925).

### Study Population

**Fig 1:**
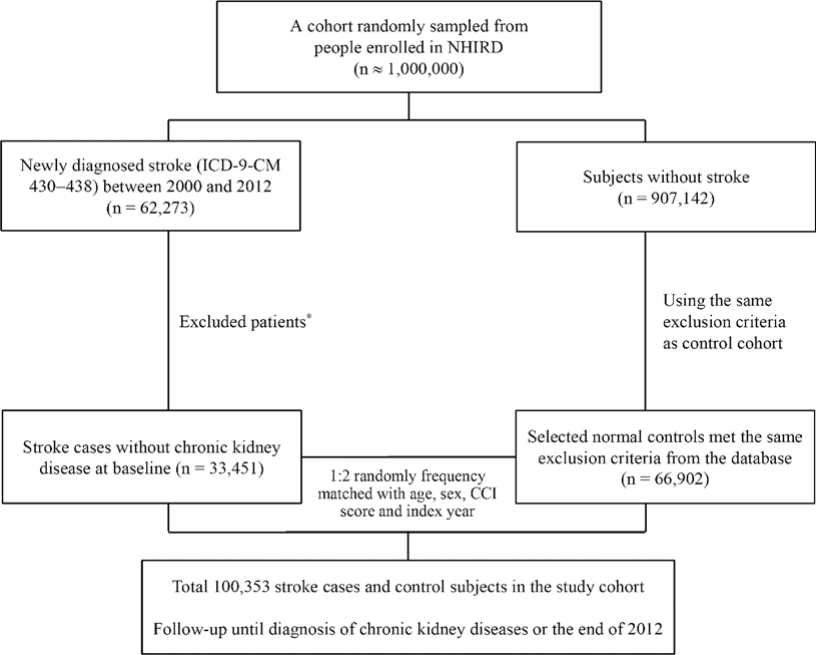
Flowchart of patient selection for the study cohort. From 2000 to 2012, 33,451 stroke patients without a history of chronic kidney diseases (CKD) or end-stage renal disease (ESRD) were identified in the National Health Insurance Research Database (NHIRD). For each stroke patient, two age-, sex- and Charlson’s comorbidity index score-matched patients in the same index year were selected from the NHIRD. *Excluding patients with CKD and ESRD before the index date (n = 10,827), those under 18 years of age (n = 379), those with missing information about age or sex (n = 458), and those who did not survive for >30 days after diagnosis of stroke (n = 210), and patients with missing matches to controls (n = 16,948).

From 1995 to 1999, we used a look-back period for identifying incident stroke patients. This 5-year look-back period was used to determine whether a patient had any prior stroke records and to reduce false incident cases. Patients without previous diagnoses of stroke (ICD-9-CM codes 430–438 and V12.54) during the look-back period were included in this study. Patients newly diagnosed with stroke (ICD-9-CM codes 430–438) from January 1, 2000, to December 31, 2012, were identified. The index date was defined as the first date of stroke diagnosis. Patients who had CKD and ESRD before the index date, those aged <18 years, those with incomplete demographic information, and those who did not survive for >30 days after diagnosis of stroke were excluded. The control cohort consisted of subjects without a history of stroke, CKD or ESRD. We used the frequency-matching method to select control subjects. For each identified stroke patient, two controls were frequency matched by age, sex, Charlson’s comorbidity index (CCI) score category, and year of index date (Fig 1).

### Outcome Measures and Relevant Variables

Outcomes and comorbidities were identified by examining ICD-9-CM codes (S1 Table). CKD was defined using ICD-9-CM codes, as previously described [17, 18]. Both the stroke and non-stroke cohorts were followed from the index date to the date CKD first occurred, the date they withdrew from the insurance system, or the end of 2012. To compare the effect of different types of stroke on CKD incidence, stroke patients were subclassified into those with ischemic and hemorrhagic stroke. Major co-morbid diseases diagnosed before the index date were defined as baseline comorbidities based on claims data. These comorbidities included hypertension, diabetes mellitus, hyperlipidemia, coronary artery disease (CAD), congestive heart failure (CHF), endocarditis, peripheral artery occlusive disease (PAOD), atrial fibrillation (AF), and gout. CCI score was used to quantify baseline comorbidities (S2 Table) [19]. To prevent double-counting and over-adjustment in multivariate regressions, CCI score did not consider stroke as a comorbidity in patients with stroke. Long-term medications thought to be associated with renal outcomes, including angiotensin-converting-enzyme inhibitors (ACEIs), angiotensin II receptor blockers (ARBs), non-steroidal anti-inflammatory drugs (NSAIDs) and Chinese herbal medicine, were also recorded. CKD progression was defined by the progression of incident CKD to advanced CKD. Advanced CKD was defined using ICD-9-CM codes and erythropoiesis-stimulating agents, as described previously [15]. ESRD was defined as undergoing dialysis therapy for at least 90 days and was identified by dialysis-related procedure codes [20].

### Statistical Analysis

Demographic and clinical characteristics in the stroke and non-stroke cohorts were summarized using proportions and mean±standard deviation (SD). x^2^ tests and *t* tests were used to compare the distributions of discrete and continuous variables, respectively. Cox’s proportional hazard models were used to estimate the relative risk of developing CKD in the stroke compared with the non-stroke cohort. Confounders, including age, sex, all comorbidities, CCI scores, visit frequency, and long-term use of medications, were adjusted in multivariate Cox’s analysis with competing risks (Fine–Gray and cause-specific hazard models) of death to estimate adjusted hazard ratios (aHRs). Because co-morbid diseases may not only be present at baseline but may develop during the follow-up period, these co-morbid diseases were modeled using non-reversible time-dependent binary covariates for the survival analyses. Additionally, multiplicative analysis was performed to assess the interactive effects of stroke and comorbidities on the risk of subsequent CKD. Multivariate Cox’s regression analysis with competing risks of death in patients with incident CKD was performed to evaluate the impact of stroke and other risk factors on progression to advanced CKD and ESRD. All variables in the survival analysis were checked for proportionality by exploratory diagnostic log-negative-log survival plots to assess the proportional hazards assumption. Interactions between survival time and covariates were investigated, and we found there were no significant interactions among these variables. Cumulative incidence of CKD, CKD progression and ESRD was calculated by the Kaplan-Meier method and compared in the stroke and non-stroke groups using log-rank tests. To assess the reliability of our results, we performed a propensity score-based sensitivity analysis for uncontrolled confounding. Propensity scores were calculated using multivariate logistic regression to predict the probability of stroke occurrence (S3 Table). We used two different propensity-score based methods: covariate adjustment using the propensity score and stratification by five strata based on propensity quintiles, to reduce bias due to unmeasured confounders [21]. Furthermore, we conducted a series of analyses defining CKD with ICD-9-CM codes within several periods of time to minimize misclassification bias, using time intervals of 90 days, 180 days, and 365 days. All statistical analyses were performed using SAS 9.4 software (SAS Institute Inc., Cary, NC). Two-tailed *P* values <0.05 were considered statistically significant.

## Results

### Characteristics of the Study Population

Fig 1 shows a flowchart for the subject selection process, and Table 1 shows the characteristics of the study population. A total of 100,353 participants were enrolled, including 33,451 patients diagnosed with stroke between January 1, 2000, and December 31, 2012 and 66,902 age-, sex-, and CCI score-matched control subjects not diagnosed with stroke. The mean follow-up times and SDs in these cohorts were 6.11±3.88 and 6.38±3.97 years, respectively. The distribution of sex and age groups was the same in both cohorts, with nearly 80% of the participants >50 years of age. Compared with the control cohort, patients with stroke were more likely to have comorbidities, such as hypertension, diabetes mellitus, hyperlipidemia, CAD, CHF, endocarditis, PAOD, AF and gout at baseline and during the follow-up period, increased visit frequency and long-term medications of ACEIs or ARBs, NSAIDs, and Chinese herbal medicine (all *P*<0.001).

**Table 1:** Comparisons in demographic characteristics, comorbidities, medications and clinical outcomes in subjects with and without stroke.

Variables^a^	Before frequency matching			Frequency matched				
	Non-stroke (n = 636,098)	Stroke (n = 50,857)	*P* value^b^	Non-stroke (n = 66,902)	Stroke (n = 33,451)	Total cohort (n = 100,353)	*P* value^b^	*StD*^c^
Sex								
Female	330,567 (51.97%)	24,221 (47.63%)	<0.001	32,104 (47.99%)	16,052 (47.99%)	48,156 (47.99%)	0.99	0.00
Male	305,530 (48.03%)	26,636 (52.37%)	<0.001	34,798 (52.01%)	17,399 (52.01%)	52,197 (52.01%)	0.99	0.00
Age, years	40.76 ± 14.95	64.53 ± 13.76	<0.001	58.71 ± 12.78	58.71 ± 12.78	58.71 ± 12.78	1.00	0.00
Age stratified								
<50	472,524 (74.28%)	7,682 (15.11%)	<0.001	14,760 (22.06%)	7,380 (22.06%)	22,140 (22.06%)	0.99	0.00
50−64	116,514 (18.32%)	16,045 (31.55%)	<0.001	29,622 (44.28%)	14,811 (44.28%)	44,433 (44.28%)	0.99	0.00
≥65	47,060 (7.4%)	27,130 (53.35%)	<0.001	22,520 (33.66%)	11,260 (33.66%)	33,780 (33.66%)	0.99	0.00
Clinic visit frequency^d^, visits per year	2.87 ± 9.59	15.75 ± 20.93	<0.001	20.05 ± 25.66	27.75 ± 25.25	22.61 ± 25.78	<0.001	0.30
Comorbidities at baseline								
Hypertension	75,033 (11.8%)	25,456 (50.05%)	<0.001	18,813 (28.12%)	15,447 (46.18%)	34,260 (34.14%)	<0.001	0.38
Diabetes mellitus	49,704 (7.81%)	14,515 (28.54%)	<0.001	11,978 (17.9%)	7,548 (22.56%)	19,526 (19.46%)	<0.001	0.12
Hyperlipidemia	86,218 (13.55%)	18,499 (36.37%)	<0.001	18,205 (27.21%)	12,114 (36.21%)	30,319 (30.21%)	<0.001	0.19
Gout	59,657 (9.38%)	11,266 (22.15%)	<0.001	10,678 (15.96%)	6,947 (20.77%)	17,625 (17.56%)	<0.001	0.12
CAD	32,589 (5.12%)	14,276 (28.07%)	<0.001	9,359 (13.99%)	7,317 (21.87%)	16,676 (16.62%)	<0.001	0.21
CHF	8,386 (1.32%)	4,590 (9.03%)	<0.001	2,462 (3.68%)	1,773 (5.3%)	4,235 (4.22%)	<0.001	0.08
AF	2,059 (0.32%)	1,830 (3.6%)	<0.001	685 (1.02%)	758 (2.27%)	1,443 (1.44%)	<0.001	0.098
Endocarditis	388 (0.06%)	170 (0.33%)	<0.001	68 (0.1%)	90 (0.27%)	158 (0.16%)	<0.001	0.04
PAOD	7,132 (1.12%)	2,320 (4.56%)	<0.001	1,676 (2.51%)	1,211 (3.62%)	2,887 (2.88%)	<0.001	0.06
Charlson’s comorbidity index score								
0	460,351 (72.37%)	22,600 (44.44%)	<0.001	37,756 (56.43%)	18,878 (56.43%)	56,634 (56.43%)	0.99	0.00
1−2	143,069 (22.49%)	21,864 (42.99%)	<0.001	26,554 (39.69%)	13,277 (39.69%)	39,831 (39.69%)	0.99	0.00
≥3	32,678 (5.14%)	6,393 (12.57%)	<0.001	2,592 (3.87%)	1,296 (3.87%)	3,888 (3.87%)	0.99	0.00
Comorbidities in the follow-up period^e^								
Hypertension	148,461 (23.34%)	42,665 (83.89%)	<0.001	35,821 (53.54%)	26,714 (79.86%)	62,535 (62.32%)	<0.001	0.58
Diabetes mellitus	97,053 (15.26%)	26,614 (52.33%)	<0.001	22,842 (34.14%)	15,432 (46.13%)	38,274 (38.14%)	<0.001	0.25
Hyperlipidemia	162,423 (25.53%)	31,720 (62.37%)	<0.001	31,877 (47.65%)	21,366 (63.87%)	53,243 (53.06%)	<0.001	0.33
Gout	95,048 (14.94%)	17,757 (34.92%)	<0.001	17,411 (26.02%)	10,963 (32.77%)	28,374 (28.27%)	<0.001	0.15
CAD	52,984 (8.33%)	23,824 (46.85%)	<0.001	15,360 (22.96%)	12,721 (38.03%)	28,081 (27.98%)	<0.001	0.33
CHF	18,979 (2.98%)	12,395 (24.37%)	<0.001	6,120 (9.15%)	5,266 (15.74%)	11,386 (11.35%)	<0.001	0.20
AF	5,763 (0.91%)	5,189 (10.2%)	<0.001	1,951 (2.92%)	2,308 (6.9%)	4,259 (4.24%)	<0.001	0.19
Endocarditis	662 (0.1%)	315 (0.62%)	<0.001	150 (0.22%)	166 (0.5%)	316 (0.31%)	<0.001	0.05
PAOD	14,641 (2.3%)	5,643 (11.1%)	<0.001	3,627 (5.42%)	3,117 (9.32%)	6,744 (6.72%)	<0.001	0.15
Long-term use of medications^f^								
ACEIs or ARBs	73,903 (11.62%)	31,062 (61.08%)	<0.001	20,432 (30.54%)	18,926 (56.58%)	39,358 (39.22%)	<0.001	0.54
NSAIDs	17,025 (2.68%)	6,872 (13.51%)	<0.001	5,732 (8.57%)	3,511 (10.5%)	9,243 (9.21%)	<0.001	0.07
Chinese herbal medicine	299,434 (47.07%)	23,895 (46.98%)	<0.001	30,875 (46.15%)	16,688 (49.89%)	47,563 (47.4%)	<0.001	0.08
Outcomes in the follow-up period								
Incident CKD	14,359 (2.26%)	7,725 (15.19%)	<0.001	3,865 (5.78%)	3,578 (10.7%)	7,443 (7.42%)	<0.001	0.18
ESRD	742 (0.12%)	532 (1.05%)	<0.001	193 (0.29%)	447 (1.33%)	640 (0.64%)	<0.001	0.12
Death	5,491 (0.86%)	3,158 (6.21%)	<0.001	1,693 (2.53%)	1,284 (3.84%)	2,977 (2.97%)	<0.001	0.07
Propensity score	0.25 ± 0.09	0.40 ± 0.13	<0.001	0.31 ± 0.12	0.38 ± 0.13	0.33 ± 0.13	<0.001	0.56

Abbreviations: ACEI, Angiotensin-converting-enzyme inhibitor; AF, atrial fibrillation; ARB, Angiotensin II receptor blocker; CAD, coronary artery disease; CHF, congestive heart failure; CKD, chronic kidney disease; ESRD, end-stage renal disease; NSAIDs, Non-steroidal anti-inflammatory drugs; PAOD, peripheral artery occlusive disease; SD, standard deviation; StD, standardized difference.

^a^Variables are expressed as Mean ± SD or n (%).

^b^2-sided *t* test or x^2^-test between stroke and non-stroke cohorts.

^c^A StD of greater than 0.1 is considered important imbalance.

^d^Outpatient and emergency department visits.

^e^Comorbidities in the follow-up period including the comorbidity at baseline and new onset of these diseases during the follow-up period.

^f^Defined as drug prescription for at least 3 consecutive months.

### Risks of Incident CKD

During the follow-up period, the proportion of patients with incident CKD (10.7% *vs*. 5.78%, *P*<0.001; Table 1) and the incidence rate of CKD (17.5 *vs*. 9.06 per 1000 person-years; Table 2) were significantly higher in the stroke than in the control cohort. After adjustment for confounders, the risk of developing CKD remained significantly higher in stroke patients (aHR, 1.43; 95% confidence interval [CI], 1.36–1.50; *P*<0.001). The incidence of CKD was higher in men than in women in both cohorts. After adjusting for confounders, the risks of CKD were similarly higher in both men (aHR, 1.46; 95% CI, 1.37–1.56; *P*<0.001) and women (aHR, 1.37; 95% CI 1.28–1.48; *P*<0.001) in the stroke than in the control cohort. The incidence rate of CKD increased with age in both cohorts. After adjusting for confounders, patients aged <50 years in the stroke cohort were at 1.6-fold higher risk of CKD than those aged <50 years in the control cohort (aHR, 1.61; 95% CI, 1.37–1.88, *P*<0.001). However, when compared to the <50 years age group, the increased risk for CKD associated with previous stroke was lower in patients aged 50–64 and ≥65 years (aHR, 1.42 and 1.35; 95% CI, 1.32–1.53 and 1.25–1.45; both *P*<0.001, respectively). Among patients without any comorbidities, those in the stroke cohort had a nearly 50% higher risk of CKD than those in the control cohort (aHR, 1.47; 95% CI, 1.33–1.62; *P*<0.001). Patients with 1–2 (aHR, 1.36; 95% CI, 1.26–1.46; *P*<0.001) and >2 (aHR, 1.48; 95% CI, 1.34–1.63; *P*<0.001) concomitant comorbidities in the stroke cohort were also at higher risk of CKD than those in the control cohort.

**Table 2:** Incidence and hazard ratios of chronic kidney disease for stroke patients compared with non-stroke cohort by demographic characteristics and comorbidities.

Variables	Subjects without stroke			Subjects with stroke			Stroke cohort *vs*. Non-stroke cohort					*P* value for interaction^a^
	Event, n	Person-years	Incidence^b^	Event, n	Person-years	Incidence^b^	cHR (95% CI)	*P* value	aHR (95% CI)Model 1^c^^,^^d^	aHR (95% CI)Model 2^c^^,^^e^	*P* value	
Overall for CKD	3,865	426,512.8	9.06 (8.78−9.35)	3,578	204,460.1	17.5 (16.93−18.07)	1.92 (1.84−2.01)	<0.001	1.43 (1.36−1.50)	1.45 (1.38−1.52)	<0.001	
Sex												0.13
Female	1,699	210,346.4	8.08 (7.69−8.46)	1,504	102,142.7	14.72 (13.98−15.47)	1.82 (1.69−1.95)	<0.001	1.37 (1.28−1.48)	1.40 (1.30−1.50)	<0.001	
Male	2,166	216,166.4	10.02 (9.6−10.44)	2,074	102,317.4	20.27 (19.4−21.14)	2.01 (1.89−2.14)	<0.001	1.46 (1.37−1.56)	1.49 (1.39−1.58)	<0.001	
Stratify age												<0.001
<50	375	102,855.6	3.65 (3.28−4.01)	468	50,101.0	9.34 (8.49−10.19)	2.53 (2.21−2.9)	<0.001	1.61 (1.37−1.88)	1.62 (1.40−1.89)	<0.001	
50−64	1,679	200,869.4	8.36 (7.96−8.76)	1,589	95,519.8	16.64 (15.82−17.45)	1.98 (1.85−2.12)	<0.001	1.42 (1.32−1.53)	1.44 (1.34−1.55)	<0.001	
≥65	1,811	122,787.7	14.75 (14.07−15.43)	1,521	58,839.2	25.85 (24.55−27.15)	1.75 (1.64−1.88)	<0.001	1.35 (1.25−1.45)	1.36 (1.27−1.46)	<0.001	
Comorbidities at baseline^f^												0.03
0	1,437	233,189.1	6.16 (5.84−6.48)	815	64,662.6	12.6 (11.74−13.47)	2.04 (1.87−2.22)	<0.001	1.47 (1.33−1.62)	1.50 (1.37−1.64)	<0.001	
1−2	1,666	145,171.4	11.48 (10.93−12.03)	1,773	100,156.8	17.7 (16.88−18.53)	1.53 (1.43−1.63)	<0.001	1.36 (1.26−1.46)	1.37 (1.28−1.47)	<0.001	
≥3	762	48,152.3	15.82 (14.7−16.95)	990	39,640.6	24.97 (23.42−26.53)	1.58 (1.43−1.73)	<0.001	1.48 (1.34−1.63)	1.49 (1.35−1.64)	<0.001	

Abbreviations: ACEI, Angiotensin-converting-enzyme inhibitor; AF, atrial fibrillation; aHR, adjusted hazard ratio; ARB, Angiotensin II receptor blocker; CAD, coronary artery disease; CCI, Charlson’s comorbidity index; CHF, congestive heart failure; cHR, crude hazard ratio; CI, confidence interval; CKD, chronic kidney disease; NSAIDs, Non-steroidal anti-inflammatory drugs; PAOD, peripheral artery occlusive disease.

^a^Multiplicative Cox hazards model.

^b^Incidence rate, per 1,000 person-years.

^c^Multivariate analysis including age, sex, comorbidities (hypertension, diabetes mellitus, hyperlipidemia, CAD, CHF, endocarditis, PAOD, AF and gout) and CCI score, visit frequency and long-term use of medications (including ACEIs, ARBs, NSAIDs and Chinese herbal medicine), where comorbidities and medications were considered time-dependent covariates.

^d^Fine and Gray competing risks regression model.

^e^Cause-specific hazards regression model.

^f^Patients with any of the comorbidities, including hypertension, diabetes mellitus, hyperlipidemia, CAD, CHF, endocarditis, PAOD, AF and gout, were classified as the comorbidity group.

We performed sensitivity analyses for risks of incident CKD. The associations between stroke and CKD remained consistent after propensity score-based adjustment (S4 Table). In addition, the estimated effects of stroke exposure were consistent with results in Table 2 no matter how we redefined the diagnostic criteria for CKD (S5 Table). Moreover, inclusion of patients with missing demographic information did not affect the results; the aHR for CKD was higher (aHR 1.44, 95% CI 1.38–1.52; *P*<0.001) in patients with stroke compared with those without stroke (S6 Table).

The interactive effects of comorbidities on incident CKD are summarized in Table 3. Patients with stroke and diabetes mellitus were at significantly higher risk of CKD than those without both (aHR, 2.12; 95% CI, 1.96–2.29; *P*<0.001). Stroke patients with concomitant hyperlipidemia or gout also had a higher risk of CKD (aHRs, 1.53 and 1.84; 95% CIs, 1.41–1.65 and 1.69–2.00; both *P*<0.001). The interactions between stroke, diabetes mellitus, hyperlipidemia, and gout were significant (*P*<0.001, *P* = 0.04, and *P* = 0.04, respectively). Besides, patients with stroke and hypertension, CAD, CHF, endocarditis, PAOD and AF were at higher risk of CKD than their matching controls (all *P*<0.05). However, the interactions between stroke and these comorbidities were not significant (all interactions *P*>0.05).

**Table 3:** Cox proportional hazards regression analysis for the risk of stroke-associated CKD with interaction of comorbidity.

Variables		No. of patients	Event, n	aHR^a^ (95% CI)	*P* value	*P* value for interaction^b^
Stroke	Diabetes mellitus					
No	No	54,924	3,076	1.00 (reference)		<0.001
No	Yes	11,978	789	1.28 (1.17−1.39)	<0.001	
Yes	No	25,903	2,571	1.31 (1.24−1.38)	<0.001	
Yes	Yes	7,548	1,007	2.12 (1.96−2.29)	<0.001	
Stroke	Hypertension					0.52
No	No	48,089	2,621	1.00 (reference)		
No	Yes	18,813	1244	1.07 (0.98−1.15)	0.119	
Yes	No	18,004	1,917	1.41 (1.32−1.50)	<0.001	
Yes	Yes	15,447	1,661	1.55 (1.43−1.67)	<0.001	
Stroke	Hyperlipidemia					0.04
No	No	48,697	2,671	1.00 (reference)		
No	Yes	18,205	1194	1.15 (1.06−1.24)	<0.001	
Yes	No	21,337	2,337	1.47 (1.39−1.56)	<0.001	
Yes	Yes	12,114	1,241	1.53 (1.41−1.65)	<0.001	
Stroke	Gout					0.04
No	No	56,224	3,018	1.00 (reference)		
No	Yes	10,678	847	1.41 (1.30−1.53)	<0.001	
Yes	No	26,504	2,702	1.46 (1.38−1.55)	<0.001	
Yes	Yes	6,947	876	1.84 (1.69−2.00)	<0.001	
Stroke	CAD					0.60
No	No	57,543	3,176	1.00 (reference)		
No	Yes	9,359	689	0.94 (0.86−1.03)	0.17	
Yes	No	26,134	2,689	1.43 (1.35−1.51)	<0.001	
Yes	Yes	7,317	889	1.31 (1.20−1.42)	<0.001	
Stroke	CHF					0.23
No	No	64,440	3,669	1.00 (reference)		
No	Yes	2,462	196	1.16 (1.00−1.35)	0.05	
Yes	No	31,678	3,374	1.40 (1.33−1.47)	<0.001	
Yes	Yes	1,773	204	1.43 (1.23−1.65)	<0.001	
Stroke	AF					0.87
No	No	66,217	3,815	1.00 (reference)		
No	Yes	685	50	1.03 (0.77−1.38)	0.84	
Yes	No	32,693	3,484	1.42 (1.35−1.49)	<0.001	
Yes	Yes	758	94	1.51 (1.22−1.86)	<0.001	
Stroke	Endocarditis					
No	No	66,834	3,857	1.00 (reference)		0.77
No	Yes	68	8	1.35 (0.68−2.66)	0.39	
Yes	No	33,361	3,562	1.42 (1.35−1.49)	<0.001	
Yes	Yes	90	16	2.18 (1.34−3.53)	0.002	
Stroke	PAOD					
No	No	65,226	3,761	1.00 (reference)		0.96
No	Yes	1,676	104	0.96 (0.79−1.17)	0.67	
Yes	No	32,240	3,450	1.42 (1.35−1.49)	<0.001	
Yes	Yes	1,211	128	1.35 (1.13−1.62)	0.001	

Abbreviations: AF, atrial fibrillation; aHR, adjusted hazard ratio; CAD, coronary artery disease; CCI, Charlson’s comorbidity index; CHF, congestive heart failure; CI, confidence interval; CKD, chronic kidney disease; PAOD, peripheral artery occlusive disease.

^a^Adjusted for age, sex, CCI score and comorbidities, visit frequency and long-term use of medications. All comorbidities and medications were considered as time-dependent covariates.

^b^Multiplicative Cox hazards model.

Table 4 shows different types of stroke associated with the relative risks and hazards of CKD. Compared with the non-stroke cohort, patients with both ischemic stroke (aHR, 1.66; 95% CI, 1.49–1.86; *P*<0.001) and hemorrhagic stroke (aHR 1.41; 95% CI, 1.34–1.48; *P*<0.001) were at significantly higher risk of developing CKD. Pairwise comparisons of aHRs revealed a higher risk of CKD in ischemic stroke patients than in hemorrhagic stroke patients (*P*<0.001, pairwise comparison).

**Table 4:** Incidence rates and hazard ratios of chronic kidney disease in patients with different types of stroke.

Variables	Event, n	Person-years	Incidence^a^ (95% CI)	cHR (95% CI)	*P* value	aHR^b^ (95% CI)	*P* value
Non-stroke	3,865	426,512.8	9.06 (8.78−9.35)	1.00 (reference)		1.00 (reference)	
Stroke							
Ischemic	3,211	181,549.8	17.69 (17.07−18.3)	1.95 (1.86−2.04)	<0.001	1.66 (1.49−1.86)	<0.001
Hemorrhagic	367	22,910.3	16.02 (14.38−17.66)	1.71 (1.53−1.90)	<0.001	1.41 (1.34−1.48)	<0.001

Abbreviations: aHR, adjusted hazard ratio; CCI, Charlson’s comorbidity index; cHR, crude hazard ratio; CI, confidence interval.

^a^Incidence rate, per 1,000 person-years.

^b^Adjusted for age, sex, CCI score and comorbidities, visit frequency and long-term use of medications. All comorbidities and medications were considered as time-dependent covariates.

**Fig 2:**
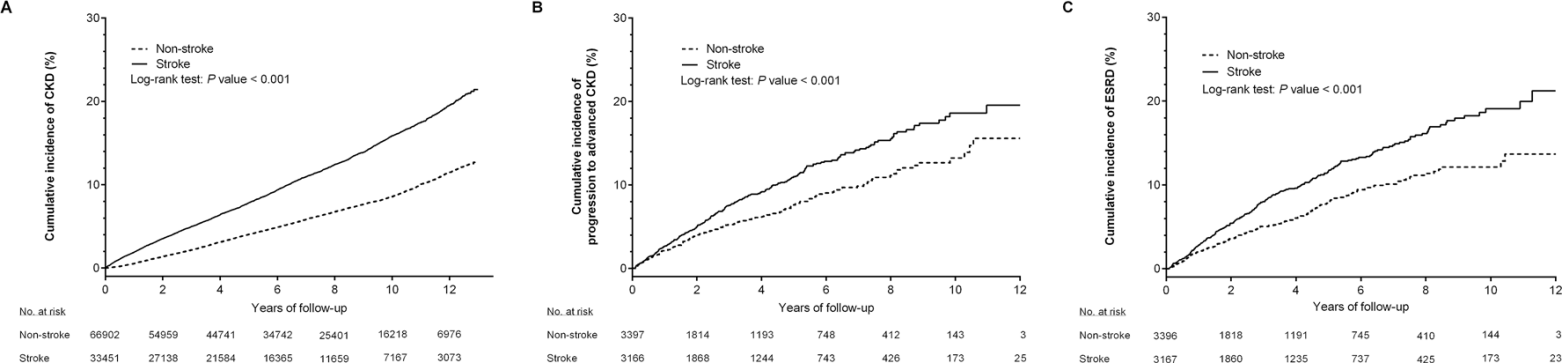
Kaplan-Meier analyses of cumulative incidence of (A) chronic kidney disease (CKD), (B) progression to advanced CKD, and (C) end-stage renal disease (ESRD) among patients with (solid line) and without (dashed line) stroke. Kaplan–Meier analyses showed that all three outcomes differed significantly for stroke and non-stroke patients, with event rates significantly higher in the stroke than in the non-stroke group (all *P*<0.001).

Kaplan–Meier survival analysis and the competing-risks cumulative incidence curve (S1A Fig) showed that cumulative incidence of CKD was significantly higher for patients in the stroke than in the non-stroke cohort (log-rank test, *P*<0.001; Fig 2A).

### Risk of CKD progression and ESRD

**Fig 3:**
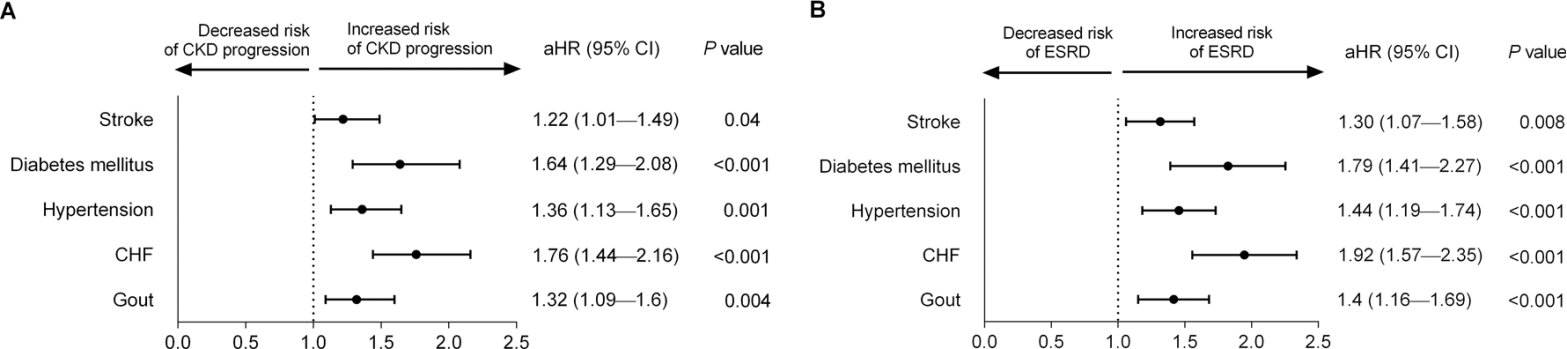
Forest plots of the associations of stroke and other significant risk factors with (A) progression to advanced chronic kidney disease (CKD) and (B) end-stage renal disease (ESRD). After adjusting for age, sex, comorbidities, Charlson’s comorbidity index score and long-term medications, stroke remained a significantly independent risk factor for CKD progression and ESRD (*P* = 0.04 and 0.008, respectively). Abbreviations: aHR, adjusted hazard ratio; CHF, congestive heart failure; CI, confidence interval.

During the follow-up period, the proportion of patients with ESRD was significantly higher in the stroke than in the control cohort (1.33% *vs*. 0.29%, *P*<0.001; Table 1). Kaplan–Meier survival analyses as well as competing-risks cumulative incidence curves (S1B and S1C Fig) showed that the cumulative incidence of CKD progression and ESRD was significantly higher for patients in the stroke than in the non-stroke group (log-rank test, both *P*<0.001) (Fig 2B and 2C). After adjusting for confounders, the risk of progression to advanced CKD remained significant for stroke patients (aHR, 1.22; 95% CI, 1.01–1.49; *P* = 0.04), as well as for subjects with diabetes mellitus, hypertension, CHF and gout (Fig 3A). Additionally, the risk of progression to ESRD was significantly higher for stroke patients (aHR, 1.30; 95% CI, 1.07–1.58; *P* = 0.008), independent of age, sex, comorbidities and long-term medications (Fig 3B).

## Discussion

In the present study, the incident rate of CKD in the stroke cohort was 17.5 per 1000 person-years, higher than the 9.06 per 1000 person-years in the control cohort. After adjusting for confounders, patients with stroke had a 1.4-fold higher risk of subsequent CKD than patients in the non-stroke cohort. Surprisingly, younger stroke patients (<50 years) were at higher risk of incident CKD than older age groups.

We observed differential relationships between comorbidity and hazards for CKD in patients with stroke compared with non-stroke patients, suggesting possible confounding effects between stroke and comorbidity on the risk of CKD. Therefore, multiplicative analysis was performed to examine the interactive effects between these factors, with significant interactions observed between stroke, diabetes mellitus, hyperlipidemia, and gout. These interactions resulted in a significant additional risk of developing CKD. The risk of CKD in patients with stroke and concomitant diabetes mellitus, hyperlipidemia or gout was higher than that in patients with stroke alone (aHRs of 2.12 *vs*. 1.31, 1.53 *vs*. 1.47 or 1.84 *vs*. 1.46).

We also examined the relationship between stroke and the risks for CKD progression and ESRD in patients with incident CKD. Kaplan–Meier analysis showed that the risks of progression to both advanced CKD and ESRD were significantly higher in patients with than without stroke (log-rank test, *P*<0.001 each). Furthermore, multivariate analyses showed that stroke was an independent factor predictive of the risks of CKD progression and ESRD.

Stroke, especially ischemic stroke, has been shown to be associated with obesity, hypertension, dyslipidemia and diabetes mellitus [22, 23]. A retrospective cross-sectional study showed that ischemic stroke was associated with higher prevalence of CKD [12], and an epidemiological study found that patients with stroke were at higher risk of ESRD [13]. To our knowledge, this study is the first and largest to show that stroke is a significant risk factor for the development of CKD, for progressive decline in renal function and for ESRD, independent of known traditional risk factors. Non-traditional risk factors may play a role in the development and progression of CKD in stroke patients; these factors may include high salt intake [24, 25], activation of the sympathetic nerve system [24, 26], neuro- or systemic inflammation [27–29], increased plasminogen activator inhibitor 1 activity [30, 31], infection [32] and oxidative stress [33].

Less is known about the association between hemorrhagic stroke and subsequent CKD [34]. In assessing the risks of subsequent CKD in patients with different types of stroke, we found that these risks were significant in patients with both ischemic and hemorrhagic stroke than in the non-stroke cohort. Furthermore, we found that the risk of CKD was higher in patients with ischemic stroke than in those with hemorrhagic stroke (aHRs of 1.66 *vs*. 1.41), suggesting that ischemic stroke patients may carry more risk factors for kidney diseases.

Cerebrorenal interactions are thought to explain the cross-talk between the brain and kidneys [7, 35]. Both organs have similar vascular beds of low resistance and high blood flow [9], as well as similar autoregulatory systems [36]. These features lead to high susceptibility to fluctuations of blood flow and pressure in the brain and kidney. Additionally, stroke and kidney diseases share common cardiovascular risk factors for small vessel diseases, including age, smoking, obesity, hypertension, diabetes mellitus and hyperlipidemia [37]. Hence, both organs are targets for arteriosclerosis or atherosclerosis. Furthermore, the linkage between cerebral and renal renin-angiotensin axes induced by high salt intake, a common risk factor of stroke [38], has been found to promote CKD progression [24]. A small-sized prospective cohort study found that silent brain ischemia was an important prognostic factor for the progression of kidney disease in CKD patients [11]. To our knowledge, this population-based study was the first to show that stroke was a strong independent predictor of the full spectrum of CKD, including the onset and progression of CKD and the development of ESRD. Stroke patients should be considered at high risk not only for disability and mortality, but for the onset and progression of CKD. Therefore, follow-up assessments of kidney function and aggressive preventive and interventional management for CKD are warranted in these patients.

The onset of stroke may indicate that cerebral arterioles had been exposed to cardiovascular risk factors or diseases for a long period of time [7]. Among the comorbidities analyzed, diabetes mellitus, hyperlipidemia and gout showed significant interactions with stroke for the onset of CKD, suggesting that diabetes, as well as hyperlipidemia and gout, may have a significant synergistic effect on the aHR for CKD. That is, diabetic stroke patients, stroke patients with hyperlipidemia or gout patients with stroke may be at greater risk of kidney diseases and require more aggressive management.

Ageing has been shown to be the leading risk factor for chronic diseases, including CKD [7, 39]. In this study, younger stroke patients were at higher risk of incident CKD than older patients. Younger patients with stroke may have a longer exposure time to stroke and other risk factors. This longer exposure may have a more pronounced effect on renal function, increasing their likelihood of kidney diseases. Additionally, younger stroke patients may have different etiologic factors that contribute to renal dysfunction, such as vasculitis and vasculopathy [13]. Moreover, stroke may occur in younger patients with more CKD risk factors or cardiovascular burdens such as smoking, hypertension, diabetes mellitus, hyperlipidemia, CHF and gout. Additionally, the induction of proinflammatory cytokines, such as interleukin-8, tumor necrosis factor-α and interleukin-6, may be more frequent in adults <50 years with a history of ischemic stroke [40]. Therefore, younger stroke patients may have more risk factors for CKD than older stroke patients.

The strengths of this study were due primarily to the use of longitudinal population-based data, representative of the general population in Taiwan. However, this study had limitations. First, the NHIRD does not include detailed information on socioeconomic status, smoking habits, family history of renal diseases, body mass index, blood pressure, lipid profile or uric acid concentrations. Additionally, the relevant biochemical parameters for evaluating CKD severity and predicting prognosis, including estimated glomerular filtration rate and albuminuria, could not be determined. Second, the number of patients with CKD and advanced CKD may have been underestimated, because study participants were enrolled mainly using ICD-9-CM codes. However, data with regard to the diagnoses of stroke, CKD, advanced CKD, ESRD and other comorbidities were reliable [14–19]. Third, results derived from a retrospective cohort study are generally of lower statistical quality than those from prospective studies because of potential biases. The higher risk of kidney diseases may be due to that stroke and CKD share similar pathophysiology rather than the ‘Brain-Kidney cross-talk’. Finally, as the majority of Taiwan’s population is of Chinese ethnicity, the findings of this study may not be applicable to populations of other ethnic backgrounds.

In conclusion, this population-based retrospective cohort study revealed that stroke is a significant and independent risk factor for CKD, renal function decline and ESRD. Careful long-term monitoring of renal function, and steps to prevent CKD, are warranted in patients with stroke and concomitant diabetes mellitus, hyperlipidemia or gout, as well as stroke patients <50 years. Because of the rising prevalence and public awareness of stroke, physicians should be aware of the risk of CKD to prevent the subsequent onset of incident CKD, advanced CKD and ESRD.

## Supporting Information

S1 Fig Cumulative incidence functions for competing risks models of (A) chronic kidney disease (CKD), (B) progression to advanced CKD, and (C) end-stage renal disease (ESRD) among patients with (solid line) and without (dashed line) stroke.
(DOCX)

S1 Table ICD-9-CM codes used to identify outcomes and comorbidities. (DOCX)

S2 Table ICD-9-CM codes and weighting of comorbidities for Charlson’s comorbidity index score. (DOCX)

S3 Table The propensity-score model results of probability of stroke. (DOCX)

S4 Table Propensity score-based sensitivity analyses for risks of incident CKD. (DOCX)

S5 Table Risks of incident CKD with respect to defining CKD at intervals of 90, 180 and 365 days. (DOCX)

S6 Table Crude and adjusted hazard ratios for chronic kidney disease without excluding patients with missing demographic information. (DOCX)
