# Supplementary material for: Stroke and Risks of Development and Progression of Kidney Diseases and End-Stage Renal Disease: A Nationwide Population-Based Cohort Study
Source: PLoS One. 2016 Jun 29;11(6):e0158533. doi: 10.1371/journal.pone.0158533 (PMC4927175; doi:10.1371/journal.pone.0158533)

**S1 Fig.** Cumulative incidence functions for competing risks models of (A) chronic kidney disease (CKD), (B) progression to advanced CKD, and (C) end-stage renal disease (ESRD) among patients with (solid line) and without (dashed line) stroke.


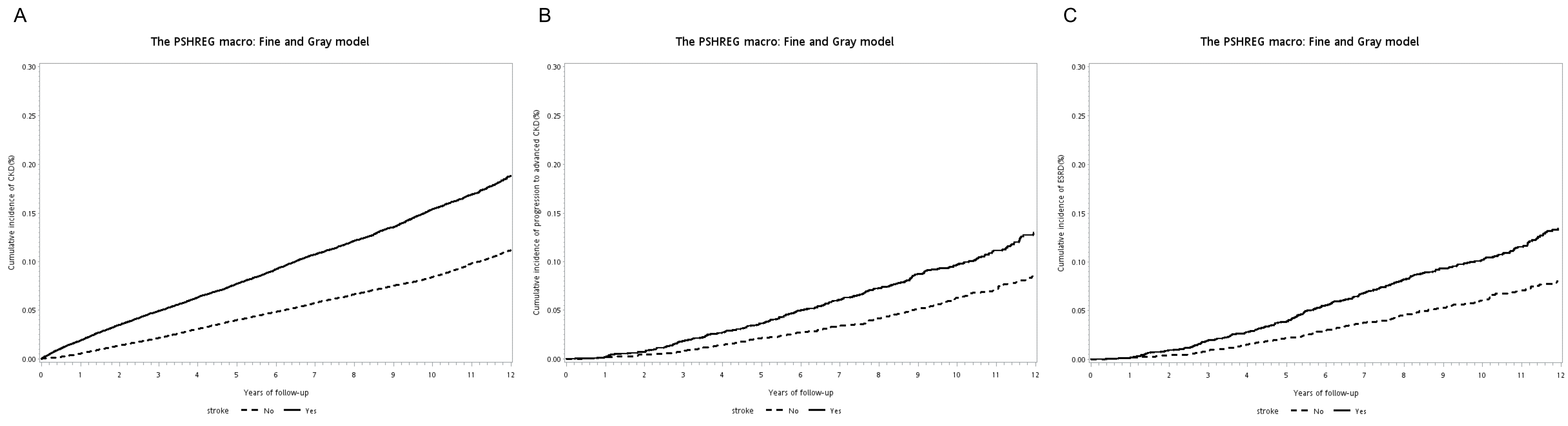

Supplement: S1 Fig — Cumulative incidence functions for competing risks models of (A) chronic kidney disease (CKD), (B) progression to advanced CKD, and (C) end-stage renal disease (ESRD) among patients with (solid line) and without (dashed line) stroke. (DOCX) [file pone.0158533.s001.docx]
