# Supplementary material for: Stroke and Risks of Development and Progression of Kidney Diseases and End-Stage Renal Disease: A Nationwide Population-Based Cohort Study
Source: PLoS One. 2016 Jun 29;11(6):e0158533. doi: 10.1371/journal.pone.0158533 (PMC4927175; doi:10.1371/journal.pone.0158533)
Supplement: S1 Table — (DOCX) [file pone.0158533.s002.docx]

**S1 Table.** ICD-9-CM codes used to identify outcomes and comorbidities.

| Diseases | Corresponding ICD-9-CM codes |
| --- | --- |
| Hemorrhagic stroke | 430.00, 431.00, 432.00, 432.90 |
| Ischemic stroke | 433.00, 433.10, 433.20, 434.00, 434.01, 434.10, 434.11, 435.00, 435.10, 435.20, 435.30, 435.90, 436.00, 437.00, 437.00, 437.10, 437.20, 437.30, 437.40, 437.50, 437.60, 437.70, 438.00, 438.00, 438.10, 438.10, 438.11, 438.12, 438.19, 438.20, 438.20, 438.21, 438.22, 438.30, 438.40, 438.50, 438.80, 438.81, 438.82, 438.83, 438.84, 438.85, 438.90 |
| Chronic kidney disease | 580.00, 580.40, 580.80, 580.81, 580.89, 580.90, 581.00, 581.10, 581.20, 581.30, 581.80, 581.81, 581.89, 581.90, 582.00, 582.10, 582.20, 582.40, 582.80, 582.80, 582.89, 582.90, 583.00, 583.10, 583.20, 583.40, 583.60, 583.70, 583.80, 583.81, 583.89, 583.90, 584.00, 584.50, 584.60, 584.70, 584.80, 584.90, 585.00, 586.00, 587.00, 588.00, 588.10, 588.90, 589.00 |
| Co-morbid diseases |  |
| Hypertension | 401–405 |
| Diabetes mellitus | 250 |
| Hyperlipidemia | 272 |
| Coronary artery disease | 410–414 |
| Congestive heart failure | 398.91, 402.01, 402.11, 402.91, 404.01, 404.03, 404.11, 404.13, 404.91, 404.93, 425.4-425.9, 428 |
| Endocarditis | 036.42, 074.22, 093.20, 093.24, 098.84, 112.81, 115.04, 115.14, 115.94, 391.10, 394.00, 397.90, 421.0–421.9, 424.9 |
| Peripheral artery occlusive disease | 443–444 |
| Atrial fibrillation | 427.31 |
| Gout | 274 |

Abbreviation: ICD-9-CM, International Classification of Disease, 9^th^ Revision, Clinical Modification.
