# Supplementary material for: Stroke and Risks of Development and Progression of Kidney Diseases and End-Stage Renal Disease: A Nationwide Population-Based Cohort Study
Source: PLoS One. 2016 Jun 29;11(6):e0158533. doi: 10.1371/journal.pone.0158533 (PMC4927175; doi:10.1371/journal.pone.0158533)
Supplement: S2 Table — (DOCX) [file pone.0158533.s003.docx]

**S2 Table.** ICD-9-CM codes and weighting of comorbidities for Charlson’s comorbidity index score

| Comorbidities | Corresponding ICD-9-CM codes | Weight |
| --- | --- | --- |
| Myocardial infarction | 410.x, 412.x | 1 |
| Congestive heart failure | 398.91, 402.01, 402.11, 402.91, 404.01, 404.03, 404.11, 404.13, 404.91, 404.93, 425.4-425.9, 428.x | 1 |
| Peripheral vascular disease | 093.0, 437.3, 440.x, 441.x, 443.1-443.9, 447.1, 557.1, 557.9, V43.4 | 1 |
| Cerebrovascular disease | 362.34, 430.x-438.x | 1 |
| Dementia | 290.x, 294.1, 331.2 | 1 |
| Chronic pulmonary disease | 416.8, 416.9, 490.x-505.x, 506.4, 508.1, 508.8 | 1 |
| Rheumatic disease | 446.5, 710.0-710.4, 714.0-714.2, 714.8, 725.x | 1 |
| Peptic ulcer disease | 531.x-534.x | 1 |
| Mild liver disease | 070.22, 070.23, 070.32, 070.33, 070.44, 070.54, 070.6, 070.9, 570.x, 571.x, 573.3, 573.4, 573.8, 573.9, V42.7 | 1 |
| Diabetes without chronic complication | 250.0-250.3, 250.8, 250.9 | 1 |
| Diabetes with chronic complication | 250.4-250.7 | 2 |
| Hemiplegia or paraplegia | 334.1, 342.x, 343.x, 344.0-344.6, 344.9 | 2 |
| Renal disease (including chronic kidney disease) | 403.01, 403.11, 403.91, 404.02, 404.03, 404.12, 404.13, 404.92, 404.93, 582.x, 583.0-583.7, 585.x, 586.x, 588.0, V42.0, V45.1, V56.x | 2 |
| Any malignancy, including lymphoma and leukemia, except malignant neoplasm of skin | 140.x-172.x, 174.x-195.8, 200.x-208.x, 238.6 | 2 |
| Moderate or severe liver disease | 456.0-456.2, 572.2-572.8 | 3 |
| Metastatic solid tumor | 196.x-199.x | 6 |
| AIDS/HIV | 042.x-044.x | 6 |

Abbreviations: AIDS, Acquired immunodeficiency syndrome; HIV, Human immunodeficiency virus; ICD-9-CM, International Classification of Disease, 9^th^ Revision, Clinical Modification.
