# Supplementary material for: Stroke and Risks of Development and Progression of Kidney Diseases and End-Stage Renal Disease: A Nationwide Population-Based Cohort Study
Source: PLoS One. 2016 Jun 29;11(6):e0158533. doi: 10.1371/journal.pone.0158533 (PMC4927175; doi:10.1371/journal.pone.0158533)
Supplement: S3 Table — (DOCX) [file pone.0158533.s004.docx]

S3 Table. The propensity-score model results of probability of stroke.

|  |  |  | 95% CI | | *P* value |
| --- | --- | --- | --- | --- | --- |
| Variables | Estimate | Odds ratio | Lower | Upper |  |
| Comorbidities at baseline |  |  |  |  |  |
| Hypertension | 0.413 | 1.511 | 1.465 | 1.559 | <0.001 |
| Diabetes mellitus | -0.097 | 0.908 | 0.875 | 0.941 | <0.001 |
| Hyperlipidemia | 0.123 | 1.131 | 1.094 | 1.168 | <0.001 |
| Gout | 0.049 | 1.05 | 1.012 | 1.089 | 0.009 |
| CAD | 0.167 | 1.181 | 1.138 | 1.226 | <0.001 |
| CHF | -0.154 | 0.857 | 0.801 | 0.917 | <0.001 |
| AF | 0.451 | 1.57 | 1.406 | 1.754 | <0.001 |
| Endocarditis | 0.649 | 1.913 | 1.377 | 2.658 | <0.001 |
| PAOD | 0.146 | 1.158 | 1.07 | 1.253 | <0.001 |
| Medications |  |  |  |  |  |
| ACEIs or ARBs | 0.907 | 2.478 | 2.406 | 2.552 | <0.001 |
| NSAIDs | 0.001 | 1.001 | 0.956 | 1.049 | 0.954 |
| Chinese herbal medicine | 0.143 | 1.153 | 1.122 | 1.185 | <0.001 |

Abbreviations: ACEI, Angiotensin-converting-enzyme inhibitor; AF, atrial fibrillation; ARB, Angiotensin II receptor blocker; CAD, coronary artery disease; CHF, congestive heart failure; CI, confidence interval; NSAIDs, Non-steroidal anti-inflammatory drugs; PAOD, peripheral artery occlusive disease.
