# Supplementary material for: Stroke and Risks of Development and Progression of Kidney Diseases and End-Stage Renal Disease: A Nationwide Population-Based Cohort Study
Source: PLoS One. 2016 Jun 29;11(6):e0158533. doi: 10.1371/journal.pone.0158533 (PMC4927175; doi:10.1371/journal.pone.0158533)
Supplement: S4 Table — (DOCX) [file pone.0158533.s005.docx]

**S4 Table.** Propensity score-based sensitivity analyses for risks of incident CKD.

| Variables | Stroke cohort *vs.* Non-stroke cohort | | | | |
| --- | --- | --- | --- | --- | --- |
|  | aHR^a^ (95% CI) | *P* value |  | aHR^b^ (95% CI) | *P* value |
| Overall for CKD | 1.50 (1.43−1.57) | <0.001 |  | 1.49 (1.42−1.57) | <0.001 |
| Sex |  |  |  |  |  |
| Female | 1.46 (1.36−1.57) | <0.001 |  | 1.45 (1.35−1.56) | <0.001 |
| Male | 1.53 (1.43−1.63) | <0.001 |  | 1.53 (1.43−1.63) | <0.001 |
| Stratify age |  |  |  |  |  |
| <50 | 1.64 (1.41−1.91) | <0.001 |  | 1.68 (1.44−1.94) | <0.001 |
| 50−64 | 1.46 (1.35−1.57) | <0.001 |  | 1.46 (1.36−1.57) | <0.001 |
| ≥65 | 1.46 (1.36−1.57) | <0.001 |  | 1.46 (1.36−1.56) | <0.001 |
| Comorbidities at baseline |  |  |  |  |  |
| 0 | 1.59 (1.48−1.70) | <0.001 |  | 1.60 (1.49−1.71) | <0.001 |
| 1−2 | 1.51 (1.41−1.62) | <0.001 |  | 1.49 (1.39−1.60) | <0.001 |
| ≥3 | 1.51 (1.23−1.85) | <0.001 |  | 1.50 (1.22−1.83) | <0.001 |
| Stratify propensity score |  |  |  |  |  |
| Q1 | − | − |  | 2.47 (2.07−2.93) | <0.001 |
| Q2 | − | − |  | 1.58 (1.35−1.84) | <0.001 |
| Q3 | − | − |  | 1.70 (1.49−1.94) | <0.001 |
| Q4 | − | − |  | 1.39 (1.30−1.50) | <0.001 |
| Q5 | − | − |  | 1.36 (1.25−1.48) | <0.001 |

Abbreviations: aHR, adjusted hazard ratio; CI, confidence interval; CKD, chronic kidney disease.

^a^Adjusted for age, sex, and propensity scores.

^b^Adjusted for age, sex, and strata by quintiles based on propensity scores.
