# Supplementary material for: Stroke and Risks of Development and Progression of Kidney Diseases and End-Stage Renal Disease: A Nationwide Population-Based Cohort Study
Source: PLoS One. 2016 Jun 29;11(6):e0158533. doi: 10.1371/journal.pone.0158533 (PMC4927175; doi:10.1371/journal.pone.0158533)
Supplement: S5 Table — (DOCX) [file pone.0158533.s006.docx]

**S5 Table.** Risks of incident CKD with respect to defining CKD at intervals of 90, 180 and 365 days

|  | Subjects without stroke | | |  | Subjects with stroke | | |  | Stroke cohort *vs.* Non-stroke cohort | | | |
| --- | --- | --- | --- | --- | --- | --- | --- | --- | --- | --- | --- | --- |
|  | Event, n | Person-years | Incidence^a^ |  | Event, n | Person-years | Incidence^a^ |  | cHR (95% CI) | *P* value | aHR^b^ (95% CI) | *P* value |
| 90 days | 2,530 | 430,056.0 | 5.88 (5.65−6.11) |  | 2,225 | 209,117.1 | 10.64 (10.2−11.08) |  | 1.82 (1.72−1.93) | <0.001 | 1.71 (1.60−1.82) | <0.001 |
| 180 days | 3,486 | 425,909.2 | 8.18 (7.91−8.46) |  | 2,733 | 206,493.4 | 13.24 (12.74−13.73) |  | 1.62 (1.54−1.71) | <0.001 | 1.53 (1.45−1.61) | <0.001 |
| 365 days | 3,697 | 408,631.3 | 9.05 (8.76−9.34) |  | 2986 | 193,015.5 | 15.47 (14.92−16.03) |  | 1.62 (1.54−1.70) | <0.001 | 1.44 (1.37−1.52) | <0.001 |

Abbreviations: ACEI, Angiotensin-converting-enzyme inhibitor; AF, atrial fibrillation; aHR, adjusted hazard ratio; ARB, Angiotensin II receptor blocker; CAD, coronary artery disease; CCI, Charlson’s comorbidity index; CHF, congestive heart failure; cHR, crude hazard ratio; CI, confidence interval; CKD, chronic kidney disease; NSAIDs, Non-steroidal anti-inflammatory drugs; PAOD, peripheral artery occlusive disease.

^a^Incidence rate, per 1,000 person-years.

^b^Adjusted for age, sex, comorbidities (hypertension, diabetes mellitus, hyperlipidemia, CAD, CHF, endocarditis, PAOD, AF and gout) and CCI score, visit frequency, and long-term use of medications (including ACEIs, ARBs, NSAIDs and Chinese herbal medicine), where comorbidities and medications were considered time-dependent covariates.
