# Supplementary material for: Stroke and Risks of Development and Progression of Kidney Diseases and End-Stage Renal Disease: A Nationwide Population-Based Cohort Study
Source: PLoS One. 2016 Jun 29;11(6):e0158533. doi: 10.1371/journal.pone.0158533 (PMC4927175; doi:10.1371/journal.pone.0158533)
Supplement: S6 Table — (DOCX) [file pone.0158533.s007.docx]

S6 Table. Crude and adjusted hazard ratios for chronic kidney disease without excluding patients with missing demographic information^a^.

|  | Subjects without stroke  (n=70,718) | | |  | Subjects with stroke  (n=33,909) | | |  | Stroke cohort *vs.* Non-stroke cohort | | | | |
| --- | --- | --- | --- | --- | --- | --- | --- | --- | --- | --- | --- | --- | --- |
|  | Event, n | Person-years | Incidence^b^ |  | Event, n | Person-years | Incidence^b^ |  | cHR (95% CI) | *P* value | aHR (95% CI)  Model 1^c,d^ | aHR (95% CI)  Model 2^c,e^ | *P* value |
| Incident CKD | 3,952 | 448,094 | 8.82 (8.54−9.09) |  | 3,672 | 207,167 | 17.72 (17.15−18.3) |  | 2.00 (1.91−2.09) | <0.001 | 1.44 (1.38−1.52) | 1.46 (1.40−1.54) | <0.001 |

Abbreviations: ACEI, Angiotensin-converting-enzyme inhibitor; AF, atrial fibrillation; aHR, adjusted hazard ratio; ARB, Angiotensin II receptor blocker; CAD, coronary artery disease; CCI, Charlson’s comorbidity index; CHF, congestive heart failure; cHR, crude hazard ratio; CI, confidence interval; CKD, chronic kidney disease; NSAIDs, Non-steroidal anti-inflammatory drugs; PAOD, peripheral artery occlusive disease.

^a^Multiple imputation was used to impute missing values.

^b^Incidence rate, per 1,000 person-years.

^c^Multivariate analysis including age, sex, comorbidities (hypertension, diabetes mellitus, hyperlipidemia, CAD, CHF, endocarditis, PAOD, AF and gout) and CCI score, visit frequency and long-term use of medications (including ACEIs, ARBs, NSAIDs and Chinese herbal medicine), where comorbidities and medications were considered time-dependent covariates.

^d^Fine and Gray competing risks regression model.

^e^Cause-specific hazards regression model.
